# Predicting mobile health clinic utilization for COVID-19 vaccination in South Carolina: A statistical framework for strategic resource allocation

**DOI:** 10.1371/journal.pgph.0003837

**Published:** 2025-06-04

**Authors:** Fatih Gezer, Kerry A. Howard, Kevin J. Bennett, Alain H. Litwin, Kerry K. Sease, Lior Rennert

**Affiliations:** 1 Department of Public Health Sciences, Clemson University, Clemson, South Carolina, United States of America; 2 Center for Public Health Modeling and Response, Clemson University, Clemson, South Carolina, United States of America; 3 University of South Carolina School of Medicine, Columbia, South Carolina, United States of America; 4 South Carolina Center for Rural & Primary Healthcare, Columbia, South Carolina, United States of America; 5 Research Center for Transforming Health, Columbia, South Carolina, United States of America; 6 Prisma Health-Upstate, Greenville, South Carolina, United States of America; 7 Clemson University School of Health Research, Clemson University, Clemson, South Carolina, United States of America; 8 University of South Carolina School of Medicine, Greenville, South Carolina, United States of America; 9 Institute for the Advancement of Community Health, Furman University, Greenville, South Carolina, United States of America; PLOS: Public Library of Science, UNITED STATES OF AMERICA

## Abstract

Mobile health clinics (MHCs) are effective tools for providing health services to disadvantaged populations, especially during health emergencies. However, patient utilization of MHC services varies substantially. Strategies to increase utilization are needed to maximize the effectiveness of MHC services by serving more patients in need. The purpose of this study is to develop a statistical framework to identify and prioritize high-risk communities for delivery of MHCs during health emergencies. Prisma Health MHCs delivered COVID-19 vaccines to communities throughout South Carolina between February 20, 2021, and February 17, 2022. In this retrospective study, we used generalized linear mixed effects models and ordinal logistic regression models to identify factors associated with, and predictive of, MHC utilization for COVID-19 vaccination by census tract. The MHCs conducted 260 visits to 149 sites and 107 census tracts. The site-level analysis showed that visits to schools (RR = 2.17, 95% CI = 1.47-3.21), weekend visits (RR = 1.38, 95% CI = 1.03-1.83), and visits when the resources were limited (term 1: 7.11, 95% CI = 4.43-11.43) and (term 2: 2.40, 95% CI = 1.76-3.26) were associated with greater MHC utilization for COVID-19 vaccination. MHC placement near existing vaccination centers (RR = 0.79, 95% CI = 0.68-0.93) and hospitals (RR = 0.83, 95% CI = 0.71-0.96) decreased utilization. Predictive models identified 1,227 (94.7%) census tracts with more than 250 individuals per MHC visit when vaccine resources were limited. Predictions showed satisfactory accuracy (72.6%). The census tracts with potential of high MHC demand had higher adolescent, 30–44 years old, and non-White populations; lower Primary Care Practitioners per 1,000 residents; fewer hospitals; and higher cumulative COVID-19 emergency department visits and deaths (compared to census tracts with low MHC demand). After the vaccines became widely available, the demand at MHCs declined. These study findings can improve MHC allocation by identifying and prioritizing medically underserved communities for strategic delivery of these limited resources, especially during health emergencies.

## Introduction

The COVID-19 pandemic has affected the lives of millions worldwide, including nearly 1.2 million deaths in the United States (US) [1]. The pandemic has also exacerbated inequalities in health outcomes, compounded with ethnic minority groups and rural communities experiencing less access for testing, vaccination, and treatment services, and greater death rates [2–8]. These consequences underscored the need for strategies to reach vulnerable communities more effectively [9,10]. Mobile health clinics (MHCs) deliver quality healthcare services to medically underserved communities who lack access to healthcare resources and facilities, especially during health emergencies [11–14]. MHCs were used during the COVID-19 pandemic for vaccination in the U.S. and different countries [15–19], and were a particular benefit to rural communities and medically underserved populations [11,12,20,21].

However, the inability to effectively identify and prioritize high-risk communities has posed daunting challenges for decision makers and has led to less-than-optimal allocation strategies [22]. This is especially problematic during phases of pandemics when resources are limited, as these phases correlate with periods of high transmission, morbidity, and mortality [23–25]. Less efficient allocation strategies have a disproportionate impact on medically underserved communities. For example, age-based allocation of COVID-19 vaccines adopted by states nationwide lead to inequity in vaccination uptake, with lower rates in economically disadvantaged neighborhoods that were at an increased risk for severe SARS-CoV-2 infection and death [26–28]. Alternatively, one study showed that the inclusion of geographic region into the prioritization process would have led to an estimated 18% decrease in COVID-19 related hospitalizations [26].

Data-driven approaches can improve emergency planning and overall health outcomes by guiding timely delivery of essential resources to high-risk communities [10,29–31]. One study showed that prioritization of COVID-19 testing to high-risk areas is twice as likely to detect positive cases compared to random allocation of tests (e.g., based on population sized) [32]. Moreover, utilization of MHC services can substantially vary by site location [33,34]. Site visits with low utilization are a missed opportunity to provide health care to individuals in need, and drastically reduce the potential impact of MHC services [22]. Therefore, projecting low- and high-demand areas at granular geographic levels can assist in optimizing the effectiveness of MHC services [35].

Various studies investigated the characteristics of individuals who used MHCs and community-level factors associated with MHC utilization [22,36,37]. Individuals who utilized the MHCs tend to be mostly ethnic minorities and uninsured persons [22,34,38]. MHCs had a higher uptake when they were located at places where the proportions of uninsured and non-white populations were higher, and primary care practitioner rates were lower [34]. Several studies built predictive models for COVID-19 vaccine uptake using structural equation models, machine learning-based approaches, and conceptual models [39–42]. However, these studies were conducted based on data for general populations rather than MHC users hence they did not target medically underserved populations. For such populations, geographic granularity is needed to effectively inform public health interventions, including MHCs [43].

In this retrospective study, we developed predictive models to predict MHC utilization for COVID-19 vaccination in South Carolina (SC) during different phases of the pandemic. The projection of MHC utilization allows us to identify the low- and high-demand census tracts for MHC utilization and understand their characteristic differences. We also explored MHC logistical factors and community determinants that contribute to greater utilization after the high-demand regions are detected, ultimately aiming to inform policy, improve public health outcomes, and optimally allocate MHCs for greater utilization, particularly during public health emergencies.

## Methods

### Setting

Prisma Health is a SC-based healthcare organization that serves 1.2 million patients per year [44]. Prisma Health deployed MHCs to increase COVID-19 vaccination in underserved communities between February 20, 2021, and February 17, 2022. A detailed explanation of Prisma Health’s MHC activities for COVID-19 vaccination program is provided in the literature [34]. All data was deidentified by Prisma Health and the study was approved by the Institutional Review Board at Clemson University (IRB2022-0150).

### Variables

Data obtained from MHC visits included the name, time, date, and location of the visit site; site type; duration of the visit; and the number of individuals who received COVID-19 vaccines. Site types include churches; public K-12 schools; universities; corporate locations such as business centers; homeless shelters; and other location types, such as community and wellness centers, supermarkets, and parks. Based on the data, we categorized the timing of the week (Monday to Thursday, Friday, or Weekend), the time of the day (morning: before 12 pm; afternoon: from 12 to 4 pm; and evening: after 4 pm), visit number (first, second, and third or more), and zip code and census tract code of the visited sites.

The community-level variables included census tract and zip code level demographic, socioeconomic, and health-related factors. Age, sex, race, ethnicity, median income, unemployment rate, labor force participation, and social vulnerability index (SVI) variables were at the census tract level and linked to census tracts of the MHC site locations. These demographic and socioeconomic variables were obtained from the United States Census Bureau American Community Survey for 2021 [45]. SVI is a measure that assesses the resilience of communities when faced with external stresses and is developed by the Agency for Toxic Substances and Disease Registry (ATSDR) at the Centers for Disease Control and Prevention (CDC) [46]. The four components of SVI are socioeconomic status, household composition and disability, minority status and language, and housing and transportation [47]. Data related to health care access were obtained from The South Carolina Center for Rural and Primary Healthcare (SCCRPH) and is available at the zip code level [48]. These variables included the number of hospitals, primary care physicians (PCP) per 1000 residents, all-cause mortality rate per 1000 residents, the percentage of uninsured individuals in each zip code, and the percentage of rural areas in each zip code. We also included the number of vaccination centers within a 2-mile radius and the number of hospitals within a 3-mile radius of each MHC location [49,50]. Data for emergency department (ED) COVID-19 hospitalizations and deaths were obtained from the South Carolina Revenue and Fiscal Affairs Office [51].

### Statistical analysis

We used negative binomial generalized linear mixed effects models to assess the relationship between site-related factors and MHC utilization for COVID-19 vaccination. For the projection of MHC utilization, we used a ranking-based approach of possible MHC utilization at the census tract level. Therefore, we used ordinal logistic regression models for the ordered categorical outcome of the MHC utilization using demographic, socioeconomic, and health-related predictors. The ordinal logistic model is adjusted for multiple variables simultaneously. Details about the model descriptions are provided in Section A in S1 Appendix.

The ordinal grouping of the MHC utilization consisted of the number of individuals with groups of 0–19, 20–49, 50–99, 100–249, 250–399, and more than 400. These groupings were used both for the validation of the models built on available MHC data, and for the projection of MHC utilization for the remaining census tracts in SC. The validation stage was performed by randomly sampling training and validation sets from the entire MHC data available for 106 census tracts. The validation set included 25 randomly sampled census tracts and the remaining data was used for the training of the models. Once the models were trained, we predicted the MHC utilization in 25 census tracts and compared it with the actual category (0–19, 20–49, 50–99, 100–249, 250–399, and more than 400) in the validation data. If the predicted category is correctly predicted meaning the predicted category is the same as the actual category in the training data for the same observation, we noted this as exact category prediction. However, if the predicted category was different than the actual category, we noted the degree of deviation by checking how many categories the prediction was away from the actual category (e.g., one group higher than the actual category or two groups lower than the actual category). We repeated the random sampling of 25 census tracts 1,000 times independently, to approximate the accuracy of the prediction performance. For the variables available at the ZCTA level, we partitioned the data into census tracts linked to that ZCTA, weighted on the census tract population.

Due to significant changes in vaccine eligibility and availability, we stratified the study period to before and after March 31, 2021. There were significant policy changes on or near these dates regarding the targeted groups for vaccination in the US. For instance, individuals aged 16 or older become eligible for COVID-19 vaccination on March 31. At the same time, COVID-19 vaccines became more widely available at pharmacies, clinics, and other health-providing organizations [52]. As a sensitivity analysis, we also stratified the time period to before and after May 10, 2021, which is the date adolescents aged 12–15 were eligible for COVID-19 vaccination [53].

## Results

### Descriptive summaries

**Fig 1:**
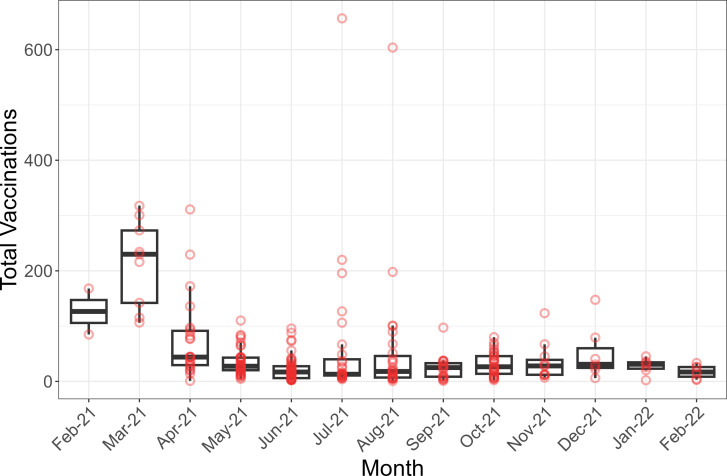
Boxplot of vaccination counts at site visits per month between February 20, 2021, and February 17, 2022. Points show the number of vaccinated individuals at each site visit for different months and constitute the box plot.

Between February 20, 2021, and February 17, 2022, the Prisma Health MHCs had 260 visits to 149 locations in SC. These visits took place in 59 zip codes (of 424 zip codes) and 107 census tracts (of 1,323 census tracts), and the MHC delivered 12,102 vaccines to 8,545 individuals. Descriptive statistics for the individuals and site types have been provided in previous research [34]. The vaccine uptake at each site visit over one-year period is shown in Fig 1. Box plots were generated based on the site visits in each month, with the number of individuals represented by the red points. MHCs had higher demand per visit before March 31, 2021, when the vaccination resources were limited and there were restrictions on certain age groups. After this date, although the MHCs increased the frequency of its activities, utilization of MHCs per visit decreased except for a school site visit that occurred in July and August 2021, in which the MHC exceeded 600 vaccinations on both visits.

**Fig 2:**
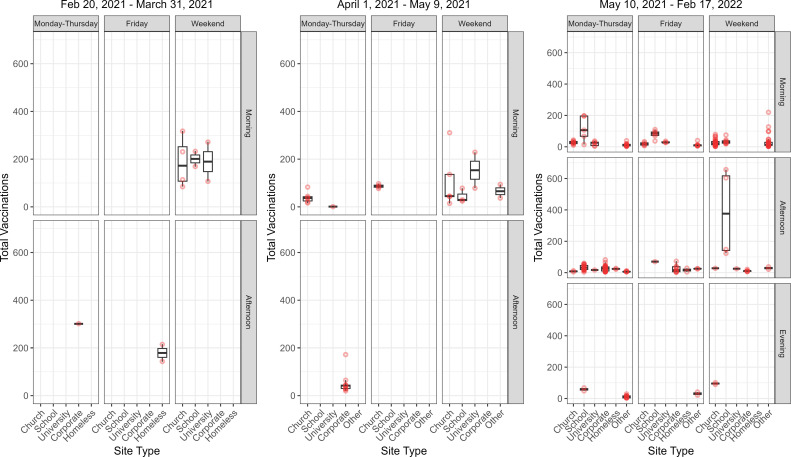
Boxplot of vaccination counts based on different terms (before March 31, 2021, between April 1 and May 9 of 2021, and after May 10, 2021), site types, the day of the week, and the time of the day.

Vaccine uptake based on the site type and the day and time of the visit for different vaccination terms (first term: before March 31, 2021; second term: between April 1 and May 9 of 2021; third term: after May 10, 2021) are summarized in Fig 2. The majority of the MHC events in the first term took place on weekends and mornings at churches, schools, and universities. Although MHCs started visits on different days and times in the second and third terms, overall MHC utilization per visit was not high especially for the third term. Considering all terms, church visits mainly occurred on weekend mornings, and school and corporate visits on Monday to Thursday afternoons.

### Site-related factors associated with MHC utilization

The site-related factors associated with MHC utilization are shown in Table 1. Estimated relative risk (RR) with 95% confidence intervals (CI) represents the relative change in the MHC utilization with respect to a standard deviation increase in the continuous predictor variables, and a category change in the categorical variables. School visits, visits on weekends, visits within the first term (between February 20, 2021, and March 31, 2021), and visits within the second term (between April 1, 2021 and February 17, 2022) were associated with higher MHC utilization. Also, MHCs showed greater utilization at their second visits to a certain site. Having nearby vaccination centers and hospitals, and types of site visits other than schools, churches, corporate locations and homeless shelters decreased MHC utilization.

**Table 1:** Results for negative binomial models that are adjusted for vaccination term, site type, day of the week, time of the day, visit number, visit duration, and population.

	RR	CI	P-value
**Vaccination Term (Ref: Third term)**			
First Term	7.11	(4.43 - 11.43)	**<0.001**
Second Term	2.40	(1.76 - 3.26)	**<0.001**
**Site type (Ref: Church)**			
School	2.17	(1.47 - 3.21)	**<0.001**
University	1.22	(0.68 - 2.17)	0.509
Corporate	1.19	(0.71 - 1.99)	0.520
Homeless	1.17	(0.49 - 2.80)	0.731
Other	0.68	(0.48 - 0.97)	**0.033**
**Day of the week (Ref: Monday – Thursday)**			
Friday	1.32	(0.97 - 1.81)	0.079
Weekend	1.38	(1.03 - 1.83)	**0.029**
**Time of the day (Ref: Morning)**			
Afternoon	1.17	(0.78 - 1.74)	0.449
Evening	1.44	(0.84 - 2.47)	0.184
**Visit number (Ref: First visit)**			
Second	1.21	(1.01 - 1.45)	**0.036**
Third or more	0.96	(0.75 - 1.25)	0.778
Visit duration	1.00	(0.89 - 1.12)	0.993
Population	1.06	(0.92 - 1.22)	0.429
Number of nearby vaccination centers	0.79	(0.68 - 0.93)	**0.003**
Number of nearby hospitals	0.83	(0.71 - 0.96)	**0.015**

### Model Validation

The agreement between the predicted and actual category of MHC utilization is summarized in Table 2 using term-based models (term 1: before March 31, 2021; term 2: after April 1, 2021). Overall, 72.6% of model predictions were within +/- 1 of the actual vaccine uptake group, as described in the methods. The models predicted the exact category of the outcome in 30.5% of the validation observations, and they predicted one group either higher or lower than the actual category for 42.1% of the validation observations.

**Table 2:** Results for prediction accuracy. Number of census tracts that have the same and deviated observed and predicted category for term-based predictions (term 1: before March 31, 2021, term 2: after April 1, 2021). Group categories for number of individuals vaccinated are 0-19, 20-49, 50-99, 100-249, 250-399, and more than 400.

	Term-based Predictions
**Predicted Category**	**N (%)**
5 groups higher than actual category	–
4 groups higher than actual category	–
3 groups higher than actual category	197 (0.8)
2 groups higher than actual category	2,598 (10.4)
**1 group higher than** actual category	**5,394 (21.6)**
**Exact group**	**7,635 (30.5)**
**1 group lower than** actual category	**5,136 (20.5)**
2 groups lower than actual category	2,974 (11.9)
3 groups lower than actual category	926 (3.7)
4 groups lower than actual category	140 (0.6)
5 groups lower than actual category	–

### Vaccine projections for census tracts

**Fig 3:**
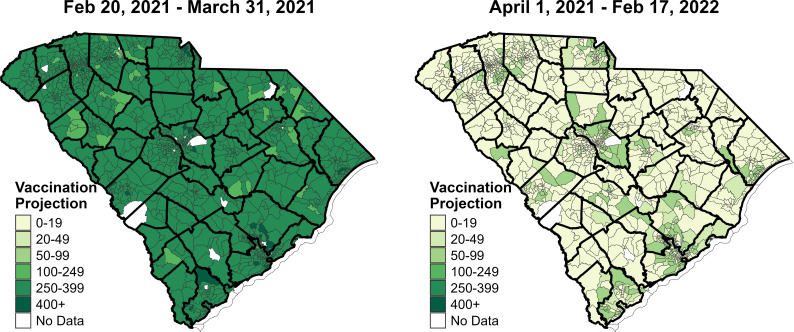
Projected MHC utilization for COVID-19 vaccination at census tracts at different time periods (pre- and post-March 31, 2021). The base map was created using the shapefiles provided in “tigris” package (https://cran.r-project.org/web/packages/tigris/) in R version 4.4.1.

We projected the MHC utilization for all census tracts of SC. These models used the characteristics of census tracts that MHCs visited as the predictors and projected the ordinal category of MHC utilization in other census tracts. The projections are performed prior and post March 31, 2021. The number of census tracts in each category of predicted MHC utilization is shown in Table 3 for both cases. Estimated coefficients for predictors, confidence intervals, p-values, and variance inflation factor (VIF) values are given in Table A in S1 Appendix. Projected MHC utilization per visit prior to March 31, 2021, was substantially higher at most census tracts (Fig 3). The bold black lines in the map show the county borders, whereas thinner lines are used for census tract borders. Census tracts are colored based on the predicted MHC demand in that area. Similar maps for predictions for different terms as sensitivity analyses are illustrated in Fig A in S1 Appendix. There were 32 (2.5%) census tracts that were projected to receive more than 400 visitors at a single MHC visit and 1,195 (92.2%) census tracts projected to receive relatively high utilization with 250–399 individuals. Only 69 (5.3%) census tracts were estimated to be utilized by under 249 individuals. However, the models predicted low overall MHC utilization starting from April 1, 2021, with 181 (14.0%) census tracts receiving between 50 and 99 individuals, and 86.0% of census tracts receiving 49 or less individuals.

**Table 3:** Number of census tracts (N) for each category of the projected MHC utilization. Projections are made for an MHC visit at any time before March 31, 2021, and after April 1, 2021.

Grouping	Before March 31	After April 1
	N = 1,296 (%)	N = 1,296 (%)
0-19	–	973 (75.0)
20-49	–	142 (11.0)
50-99	4 (0.3)	181 (14.0)
100-249	65 (5.0)	–
250-399	1,195 (92.2)	–
400+	32 (2.5)	–

Census tracts categorized in the highest and lowest MHC utilization categories had substantial differences in some of their community-level characteristics (Table 4). Compared to the low MHC utilization category, the highest category census tracts had a higher proportion of individuals under 18 years of age and 30–44 age group and a higher proportion of non-White and Hispanic individuals. These census tracts had lower PCP rates, lower number of hospitals, higher cumulative COVID-19 ED visits and deaths prior to when MHCs were deployed on February 20, 2021. Sensitivity analyses for the cutoff date of May 10, 2021, and for 12-month term are provided Tables B-G in S1 Appendix. The distributions of each predictor variable in the training sites (i.e., locations with data where MHCs visited) and the predicted sites (i.e., locations without data where MHCs did not visit) sufficiently overlap (Fig B in S1 Appendix).

**Table 4:** Characteristics of census tracts that are projected to have high (groups: 250-399 and 400+) and low (groups: 50-99 and 100-249) MHC utilization for COVID-19 vaccination before March 31, 2021. Median values with IQR and p-values for significance difference in the medians are provided.

	High Utilization	Low Utilization	P-value
	N = 1,227	N = 69	
% Age under 18	21.6 (18.0-25.1)	20.1 (13.9-23.4)	**0.044**
% Age 18–29	14.1 (11.0-17.5)	16.3 (12.6-34.7)	**0.001**
% Age 30–44	18.3 (15.0-21.5)	16.2 (11.5-19.7)	**0.011**
% Age 45–64	26.5 (23.0-29.9)	25.7 (16.9-31.0)	0.263
% Age over 65	17.4 (13.4-21.8)	16.7 (11.9-21.7)	0.398
% Male	48.5 (45.9-51.1)	49.8 (46.9-53.0)	**0.030**
% Non-white	35.6 (20.5-55.1)	25.8 (9.1-37.5)	**0.009**
% Hispanic	4.8 (2.8-7.9)	3.7 (2.7-5.9)	**0.022**
SVI	0.5 (0.3-0.8)	0.6 (0.3-0.8)	0.053
Income (×$1000)	55.3 (42.6-71.3)	46.9 (34.0-62.6)	**0.007**
% Unemployed	4.6 (2.6-7.6)	4.2 (2.6-6.9)	**<0.001**
PCP rate	0.3 (0.0-0.8)	3.9 (0.5-16.0)	**<0.001**
Hospitals	0.0 (0.0-1.0)	0.9 (0.2-1.5)	**<0.001**
% Uninsured	10.9 (8.8-12.3)	10.2 (7.7-11.6)	0.426
Mortality rate	51.3 (40.7-60.7)	50.3 (38.3-60.3)	0.797
% in Poverty	11.0 (7.1-15.7)	14.0 (10.2-17.3)	**0.006**
% in Rural	28.0 (5.0-60.0)	15.0 (0.2-62.4)	0.102
COVID-19 ED visits	1,189 (658-2,012)	846 (504-1,260)	**0.002**
COVID-19 deaths	64 (35-99)	41 (22-70)	**0.002**

## Discussion

This study aims to serve as a tool to improve the MHCs’ activities. The study provides important insights from the utilization of MHCs during COVID-19 vaccination across South Carolina, highlighting the potential to optimize resource allocation by identifying high-uptake areas and understanding the factors influencing the success of MHCs. The findings underscore the importance of strategically deploying MHCs to maximize vaccine reach, particularly among underserved and vulnerable populations.

Operational factors including site type (e.g., church, school, etc.), day, and time of the week were significantly associated with vaccine uptake. Notably, visits to schools and visits conducted on weekends were linked to higher vaccine uptake. During the pre- and post-March 31, 2021, 72.7% and 38.6% of MHC visits were conducted on weekends with median utilization of 199 (IQR: 112–243) and 23 (IQR: 10 – 38) per visit, respectively.[34] Although the school visits were similar for pre- and post-March 31, 2021 with 18.2% and 18.5%, the utilization per visit was 200 (IQR: 184–217) and 45 (IQR: 24–77) making them the highest utilized location for post-March 31, 2021, followed by universities 27 (IQR: 17–36). These findings suggest that MHCs can achieve a more significant impact by targeting educational institutions and scheduling visits on days and times that are more convenient for community members. Moreover, the presence of nearby vaccination centers and hospitals was found to negatively impact MHC utilization. Therefore, that MHCs are particularly valuable in areas with limited access to fixed-site vaccination centers, highlighting the importance of strategic placement in underserved regions.

The predictive models identified 1,227 census tracts with higher potential for vaccine uptake. These areas had a higher rate of uninsurance and mortality, a higher proportion of ethnic minority populations and adolescents, and were in rural areas. These findings are consistent with MHC-centered studies conducted for other states and cities [36–38],

We also found healthcare-related factors associated with MHC utilization. Census tracts with lower rates of primary care practitioners, hospitals, and other vaccination sites had higher MHC utilization compared to the low-demand census tracts, highlighting the potential of MHC utilization at locations with limited healthcare resources and facilities. The role of predictive models is to act as a classifying or categorization mechanism that identifies areas and communities that are more likely to use the MHCs given the limited data from previous MHC visits. A possible setting for MHC allocation could be to use the existing data and base allocation on the categorical projection of new areas, such as census tracts where the MHCs could possibly get high demand. As MHCs conduct visits to more places relying on the projected high-demand areas, the models can be validated and updated based on the new data. Hence this framework can allow for improving optimal deployment of MHCs to gain the highest utilization, especially by individuals and communities who need these services the most.

The time component of projections for MHC utilization at the census tract level provides important decision-making implications for public health planning. Before March 31, 2021, the models predicted high MHC utilization in most census tracts, reflecting the high demand for vaccines during the initial rollout phase. However, after this date, the predicted utilization decreased significantly, indicating the need for continuous assessment and adaptation of MHC deployment strategies as the pandemic evolves and vaccine availability at other providers changes.

This study has several limitations. First, there were substantial changes in vaccine eligibility and availability which affected the MHC utilization. The retrospective design and reliance on limited data may not capture all relevant factors influencing MHC utilization. These factors may also differ from state to state. Future research should consider prospective studies and incorporate additional factors such as community engagement and outreach efforts to provide a more comprehensive understanding of MHC effectiveness. To extend the similar framework to other states, countries, and to different diseases, one would need to consider the determinants of the regions and the disease. We were able to assess model accuracy and generalizability by comparing predictor distributions between training and prediction sites, finding substantial overlap that supports positivity and exchangeability. However, since the primary aim of this study was to develop a predictive model rather than establishing causal relationships, we did not explicitly address the stable unit treatment value assumption. Future research can also improve this analytic framework through variable selection or incorporation of additional variables to improve prediction accuracy.

In conclusion, this study provides a framework for optimizing the deployment of MHCs in future health emergencies by identifying factors associated with higher vaccine uptake and predicting areas of high utilization. Predicting the highest- and lowest-demand census tracts for MHC utilization provides categorized importance structure for public health planning and resource allocation. Strategic allocation of MHCs based on these insights can enhance the timely delivery of essential resources to the most vulnerable communities during health emergencies and ultimately save more lives.

## Supporting information

S1 Appendix Supplementary methods, figures and tables. (DOCX)
