# Supplementary material for: Predicting mobile health clinic utilization for COVID-19 vaccination in South Carolina: A statistical framework for strategic resource allocation
Source: PLOS Glob Public Health. 2025 Jun 4;5(6):e0003837. doi: 10.1371/journal.pgph.0003837 (PMC12136404; doi:10.1371/journal.pgph.0003837)
Supplement: S1 Appendix — (DOCX) [file pgph.0003837.s001.docx]

**Predicting Mobile Health Clinic Utilization for COVID-19 Vaccination in South Carolina: A Statistical Framework for Strategic Resource Allocation**

**S1 Appendix**

**Table of Contents**

**Section A: Supplementary Methods**  1

Negative binomial generalized linear mixed effects model. 1

Ordinal logistic regression model. 1

**Section B: Supplementary Figures** 3

**Fig A:** Projected MHC utilization for COVID-19 vaccination at census tracts at different time periods (12-months, and pre and post May 10, 2021). The base map was created using the shapefiles provided in “tigris” package (https://cran.r-project.org/web/packages/tigris/) in R version 4.4.1. 3

**Fig B:** Overlaid density curves for variables used in the prediction model. Red line shows the density based on census tracts to train the model, and black line shows the density of census tracts in the prediction data for each variable. The x-axis shows the variable values and y-axis shows the estimated density values. 4

**Section C: Supplementary Tables** 5

**Table A:** Results for the prediction model. Estimated relative risk (RR), confidence interval (CI), p-value and variance inflation factor (VIF) are given for each predictor. 5

**Table B**: Characteristics of census tracts that are projected to have high (group: 50-99) and low (groups: 0-19 and 20-49) MHC utilization for COVID-19 vaccination after April 1, 2021. Median values with IQR and p-values for significance of the difference of medians are provided. 6

**Table C**: Results for prediction accuracy. Number of census tracts that have the same and deviated observed and predicted category for 12-month predictions, and term-based predictions (term 1: before May 9, 2021, term 2: after May 10, 2021). 7

**Table D**: Number of census tracts (N) for each category of the projected MHC utilization. Projections are made for an MHC visit at any time during 12-month period (February 20, 2021, to February 17, 2022), before May 9, 2021, and after May 10, 2021. 8

**Table E**: Characteristics of census tracts that are projected to have high (groups: 50-99, 100-249, and 400+) and low (groups: 0-19 and 20-49) MHC utilization for COVID-19 vaccination before May 9, 2021. Median values with IQR and p-values for significance of the difference of medians are provided. 9

**Table F**: Characteristics of census tracts that are projected to have high (groups: 20-49, 50-99, and 100-249) and low (group: 0-19) MHC utilization for COVID-19 vaccination after May 10, 2021. Median values with IQR and p-values for significance of the difference of medians are provided. 10

**Table G**: Characteristics of census tracts that are projected to have high (groups: 20-49, 50-99, 100-249, and 250-399) and low (group: 0-19) MHC utilization for COVID-19 vaccination any time during 12-month period. Median values with IQR and p-values for significance of the difference of medians are provided. 11

**Section A: Supplementary Methods**

**Negative binomial generalized linear mixed effects model:**

$$log(\mu_{ij})= \mathbf{X}_{ij}^{T}\beta+ b_{j}+ c_{ij}$$

The model assumes the following conditions:

$\boldsymbol{Y}_{\boldsymbol{ij}}$: Number of individuals utilized the mobile health clinic (MHC) at i-th site visit in the j-th census tract. The outcome variable $Y_{ij}$ follows the negative binomial distribution $Y_{ij}\sim NegBin(\mu_{ij}, \theta)$ where $\mu_{ij}$ is the mean of the negative binomial distribution at i-th site visit in the j-th census tract and $\theta$ is the dispersion parameter.

$\mathbf{X}_{\boldsymbol{ij}}^{\mathbf{T}}$: Vector of fixed effects for the i-th site visit in the j-th census tract. Fixed effects included the census tract population, visit term (term 1: before March 31, 2021, and after March 31, 2021), site category (food banks, schools, universities, corporate, homeless shelters, and other), time of the week (Monday to Thursday, Friday, and weekend), time of the week (morning, afternoon, and evening), visit number (first, second, and third or more), and the duration of the visit. The number of vaccination centers and hospitals close to the MHC location is added to the model separately to avoid collinearity between these variables. The visit time changed to (term 1: before May 10, 2021, and after May 10, 2021) for the sensitivity analysis.

$\boldsymbol{b}_{\boldsymbol{j}}$: Random effect for the j-th census tract with $b_{j}\sim N(0, \sigma_{b}^{2})$

$\boldsymbol{c}_{\boldsymbol{ij}}:$ Random effect for the i-th site in the j-th census tract $c_{ij}\sim N(0, \sigma_{b}^{2})$

**Ordinal logistic regression model**

Let Y be the ordered categorical outcome of number of individuals utilized the MHC in a census tract {1: 10-19, 2: 20-49, 3: 50-99, 4: 100-249, 5: 250-399, and 6: more than 400 individuals}. Ordinal logistic regression calculates the logged odds of being equal or less than a specific category k of the outcome variable. The cumulative logit model (proportional odds model) can be expressed as:

$$\log\frac{P(Y_{i}\leq k|X_{i})}{1-P(Y_{i}\leq k|X_{i})}= \alpha_{k}-\mathbf{X}_{i}^{T}\beta$$

$Y_{i}$: The ordered categorical outcome {1: 10-19, 2: 20-49, 3: 50-99, 4: 100-249, 5: 250-399, and 6: more than 400 individuals} of MHC for i-th census tract.

$\mathbf{X}_{i}^{T}$: The vector of fixed effects for the i-th census tract. These effects include vaccination term (first term: Feb 20, 2021 to March 31, 2021 and second term: April 1, 2021 to Feb 17, 2021) for the original analysis and (first term: Feb 20, 2021 to May 9, 2021 and second term: May 10, 2021 to Feb 17, 2021) for the sensitivity analysis, number of visits conducted to the census tract, census tract level population, proportion of individuals within 30-44, 45-64, and 65+ years of ages, proportion of males, non-White, unemployed, labor force participation, uninsured, under poverty, social vulnerability index (SVI), median income, primary care physicians per 1,000 people (PCP rate), all-cause mortality rate, hospital presence, and percent of rural areas. Variables that are available for zip codes are transformed to census tract level by weighting on the population of a census tract living in different zip codes.

Finally, $\alpha_{k}$ are the threshold parameters (cut-points) separating the adjacent categories of the ordinal response.

**Section B: Supplementary Figures**


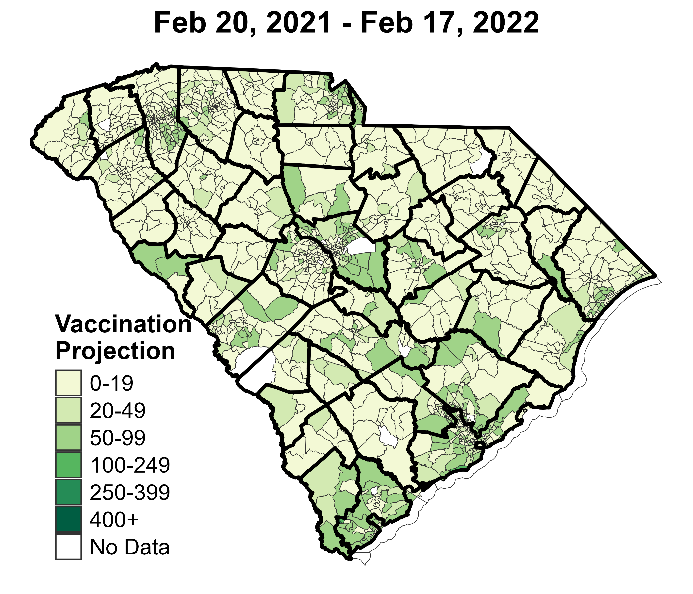


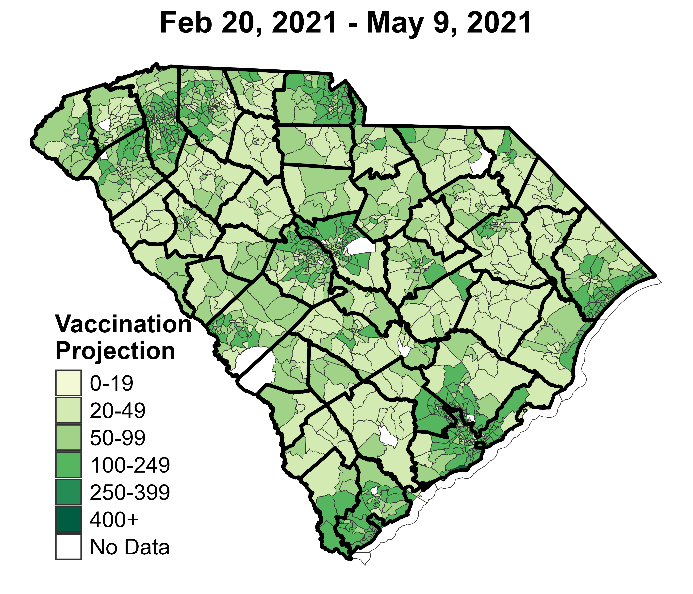


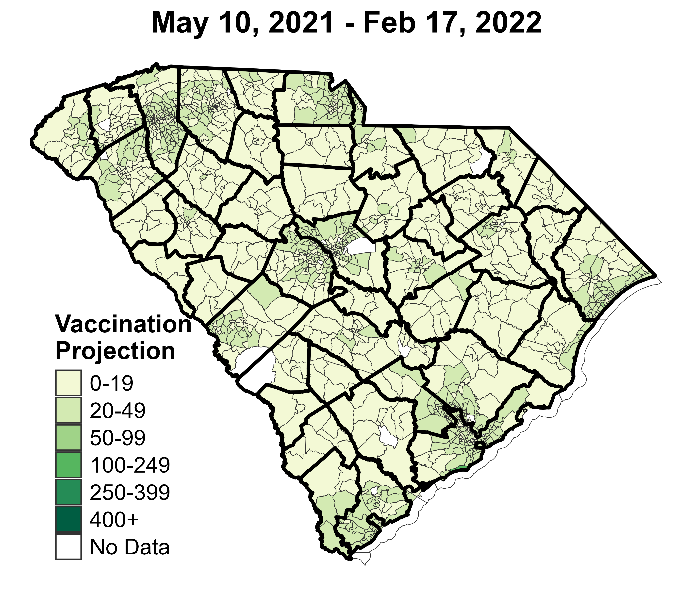


**Fig A**: Projected MHC utilization for COVID-19 vaccination at census tracts at different time periods (12-months, and pre and post May 10, 2021). The base map was created using the shapefiles provided in “tigris” package (<https://cran.r-project.org/web/packages/tigris/>) in R version 4.4.1.


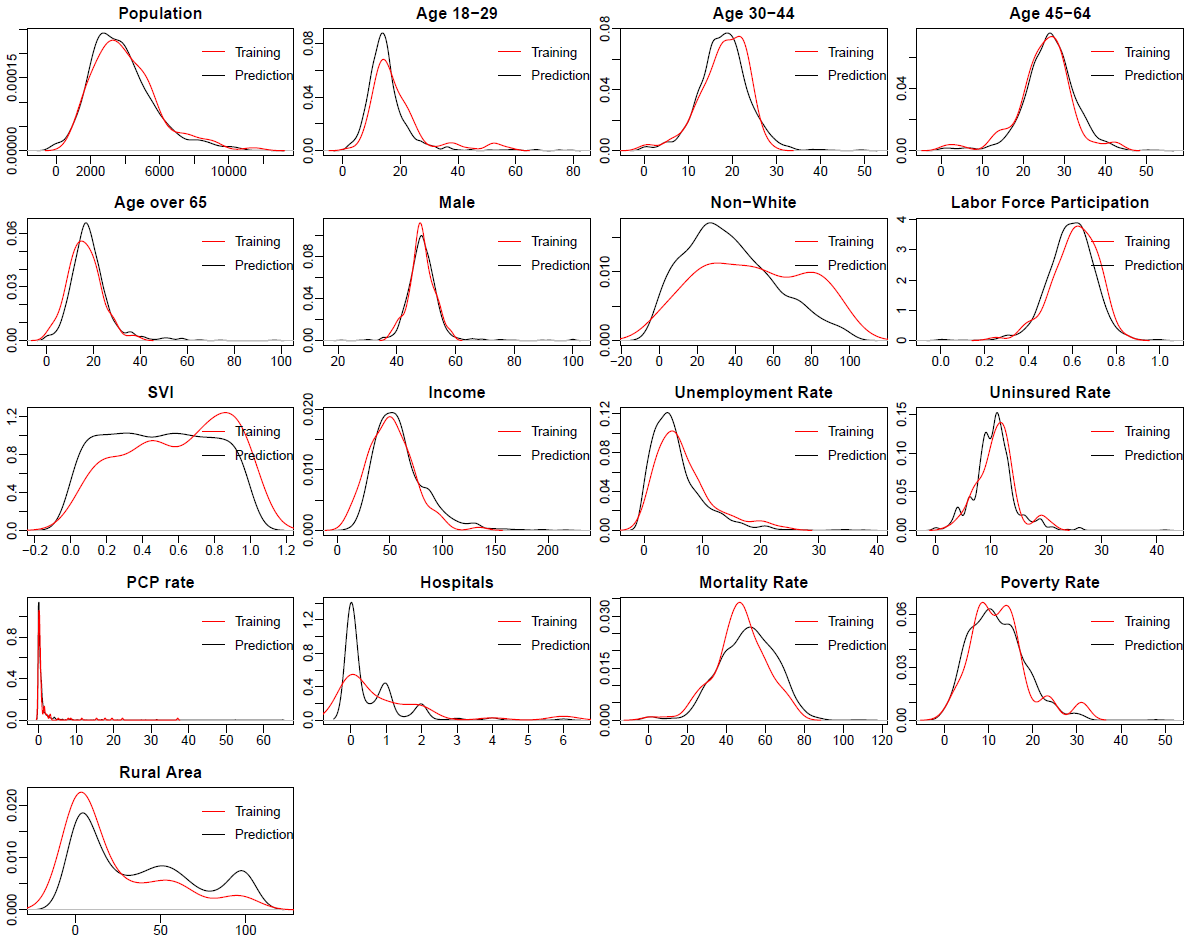


**Fig B**: Overlaid density curves for variables used in the prediction model. Red line shows the density based on census tracts to train the model, and black line shows the density of census tracts in the prediction data for each variable. The x-axis shows the variable values and y-axis shows the estimated density values.

**Section C: Supplementary Tables**

**Table A:** Results for the prediction model. Estimated relative risk (RR), confidence interval (CI), p-value and variance inflation factor (VIF) are given for each predictor.

| **Variable** | **Estimate** | **CI** | **P-value** | **VIF** |
| --- | --- | --- | --- | --- |
| First term (**ref:** second term) | 210.33 | (43.45 - 1168.39) | **<0.001** | 1.49 |
| Number of visits | 2.30 | (1.76 - 3.11) | **<0.001** | 1.49 |
| Census tract population | 1.43 | (0.92 - 2.27) | 0.115 | 1.66 |
| % Age 30-44 (**ref:** % Age 18-29) | 1.04 | (0.63 - 1.70) | 0.886 | 2.07 |
| % Age 45-64 (**ref:** % Age 18-29) | 1.11 | (0.68 - 1.84) | 0.677 | 1.95 |
| % Age over 65 (**ref:** % Age 18-29) | 1.17 | (0.70 - 1.94) | 0.544 | 2.33 |
| % Male (**ref:** % Female) | 0.99 | (0.65 - 1.51) | 0.974 | 1.58 |
| % Non-white (**ref:** % White) | 1.66 | (0.92 - 3.02) | 0.090 | 3.10 |
| % Unemployed | 1.14 | (0.73 - 1.81) | 0.559 | 1.89 |
| Labor force participation | 1.19 | (0.65 - 2.19) | 0.574 | 3.29 |
| SVI | 0.76 | (0.40 - 1.45) | 0.406 | 3.76 |
| Median household income | 1.08 | (0.57 - 2.11) | 0.824 | 3.80 |
| PCP rate | 0.75 | (0.45 - 1.18) | 0.223 | 1.47 |
| % Uninsured | 1.10 | (0.65 - 1.83) | 0.727 | 2.17 |
| Mortality rate | 1.01 | (0.56 - 1.84) | 0.975 | 3.03 |
| Poverty rate | 0.81 | (0.46 - 1.39) | 0.436 | 2.58 |
| % Rural area | 1.09 | (0.70 - 1.71) | 0.694 | 1.76 |
| Hospital presence | 0.82 | (0.54 - 1.23) | 0.331 | 1.45 |

**Table B**: Characteristics of census tracts that are projected to have high (group: 50-99) and low (groups: 0-19 and 20-49) MHC utilization for COVID-19 vaccination after April 1, 2021. Median values with IQR and p-values for significance of the difference of medians are provided.

|  | **High Utilization**  **N = 181** | **Low Utilization**  **N = 1,115** | **P-value** |
| --- | --- | --- | --- |
| % Age under 18 | 21.3 (17.7-25.3) | 21.6 (18.0-25.0) | 0.480 |
| % Age 18-29 | 13.4 (10.5-16.0) | 14.5 (11.4-18.1) | **0.002** |
| % Age 30-44 | 19.3 (15.9-22.6) | 17.7 (14.7-20.9) | **<0.001** |
| % Age 45-64 | 27.3 (23.6-30.6) | 26.3 (22.5-29.7) | **0.006** |
| % Age over 65 | 16.0 (12.7-21.2) | 17.7 (14.1-22.0) | **<0.001** |
| % Male | 48.5 (46.1-50.8) | 48.5 (45.9-51.4) | 0.862 |
| % Non-white | 35.1 (23.0-54.4) | 34.8 (18.9-54.3) | 0.949 |
| % Hispanic | 5.7 (3.6-8.3) | 4.5 (2.6-7.4) | **<0.001** |
| SVI | 0.29 (0.14-0.53) | 0.57 (0.32-0.80) | **<0.001** |
| Income (×$1000) | 69.8 (55.7-90.1) | 50.5 (39.3-63.2) | **<0.001** |
| % Unemployed | 4.3 (2.2-6.6) | 4.8 (2.7-8.0) | **<0.001** |
| PCP rate | 0.2 (0.0-0.6) | 0.4 (0.1-0.9) | **<0.001** |
| Hospitals | 0.0 (0.0-0.2) | 0.1 (0.0-1.0) | **<0.001** |
| % Uninsured | 9.2 (7.0-11.6) | 11.0 (9.0-12.5) | **<0.001** |
| Mortality rate | 42.3 (33.6-50.1) | 54.0 (44.6-63.2) | **<0.001** |
| % in Poverty | 7.7 (4.8-11.2) | 12.6 (8.8-16.6) | **<0.001** |
| % in Rural | 10.6 (1.8-44.7) | 34.1 (6.8-64.4) | **<0.001** |
| Hospitalizations | 1,271 (796-2,014) | 1,106 (640-1,980) | 0.072 |
| Dead | 57 (32-92) | 64 (33-100) | **0.050** |

**Table C**: Results for prediction accuracy. Number of census tracts that have the same and deviated observed and predicted category for 12-month predictions, and term-based predictions (term 1: before May 9, 2021, term 2: after May 10, 2021).

|  | **12-Month Predictions** | **Term-based Predictions** |
| --- | --- | --- |
| **Predicted Category** | **N (%)** | **N (%)** |
| 5 groups higher than truth | - | 18 (0.1) |
| 4 groups higher than truth | 4 (0.0) | 11 (0.0) |
| 3 groups higher than truth | 286 (1.1) | 409 (1.6) |
| 2 groups higher than truth | 2,678 (10.7) | 1,841 (7.4) |
| **1 group higher than truth** | **5,110 (20.4)** | **5,898 (23.6)** |
| **Exact group** | **7,158 (28.6)** | **6,539 (26.2)** |
| **1 group lower than truth** | **4,869 (19.5)** | **5,495 (22.0)** |
| 2 group lower than truth | 3,069 (12.3) | 3362 (13.4) |
| 3 group lower than truth | 1,402 (5.6) | 1,213 (4.9) |
| 4 group lower than truth | 365 (1.5) | 214 (0.9) |
| 5 group lower than truth | 47 (0.2) | - |

**Table D**: Number of census tracts (N) for each category of the projected MHC utilization. Projections are made for an MHC visit at any time during 12-month period (February 20, 2021, to February 17, 2022), before May 9, 2021, and after May 10, 2021.

| **Grouping** | **12-Month** | **Before**  **May 9** | **After**  **May 10** |
| --- | --- | --- | --- |
|  | **N = 1,296 (%)** | **N = 1,296 (%)** | **N = 1,296 (%)** |
| 0-19 | 914 (70.5) | 5 (0.4) | 753 (58.1) |
| 20-49 | 187 (14.4) | 388 (29.9) | 540 (41.7) |
| 50-99 | 192 (14.8) | 455 (35.1) | 2 (0.2) |
| 100-249 | 2 (0.2) | 447 (34.5) | 1 (0.1) |
| 250-399 | 1 (0.1) | - | - |
| 400+ | - | 1 (0.1) | - |

**Table E**: Characteristics of census tracts that are projected to have high (groups: 50-99, 100-249, and 400+) and low (groups: 0-19 and 20-49) MHC utilization for COVID-19 vaccination before May 9, 2021. Median values with IQR and p-values for significance of the difference of medians are provided.

|  | **High Utilization**  **N = 903** | **Low Utilization**  **N = 393** | **P-value** |
| --- | --- | --- | --- |
| % Age under 18 | 21.0 (17.5-24.6) | 22.7 (18.8-26.1) | **<0.001** |
| % Age 18-29 | 13.9 (10.7-17.7) | 14.8 (12.3-18.1) | **0.007** |
| % Age 30-44 | 18.5 (15.2-21.8) | 17.5 (14.2-20.9) | **0.009** |
| % Age 45-64 | 27.1 (23.6-30.5) | 25.1 (21.0-28.6) | **<0.001** |
| % Age over 65 | 17.1 (13.2-22.0) | 17.9 (14.2-21.7) | 0.122 |
| % Male | 48.9 (46.6-51.6) | 47.4 (44.6-50.4) | **<0.001** |
| % Non-white | 31.5 (18.0-51.0) | 39.8 (25.3-59.1) | **<0.001** |
| % Hispanic | 5.4 (3.4-8.5) | 3.1 (2.0-5.8) | **<0.001** |
| SVI | 0.4 (0.2-0.7) | 0.6 (0.4-0.8) | **<0.001** |
| Income (×$1000) | 61.2 (47.8-78.9) | 43.1 (34.8-53.7) | **<0.001** |
| % Unemployed | 4.5 (2.3-7.0) | 5.1 (3.1-8.4) | **<0.001** |
| PCP rate | 0.3 (0.0-0.7) | 0.5 (0.1-1.5) | **<0.001** |
| Hospitals | 0.0 (0.0-1.0) | 0.2 (0.0-1.0) | **0.001** |
| % Uninsured | 10.7 (8.3-12.6) | 10.7 (9.0-11.8) | 0.727 |
| Mortality rate | 48.0 (38.6-56.8) | 58.7 (49.6-66.1) | **<0.001** |
| % in Poverty | 10.1 (6.1-14.5) | 14.4 (10.6-18.4) | **<0.001** |
| % in Rural | 14.9 (2.4-45.7) | 59.7 (33.2-92.5) | **<0.001** |
| COVID-19 ED visits | 1,339 (796-2,091) | 843 (430-1,432) | **<0.001** |
| COVID-19 deaths | 67 (40-104) | 46 (22-76) | **<0.001** |

**Table F**: Characteristics of census tracts that are projected to have high (groups: 20-49, 50-99, and 100-249) and low (group: 0-19) MHC utilization for COVID-19 vaccination after May 10, 2021. Median values with IQR and p-values for significance of the difference of medians are provided.

|  | **High Utilization**  **N = 543** | **Low Utilization**  **N = 753** | **P-value** |
| --- | --- | --- | --- |
| % Age under 18 | 20.8 (17.4-24.4) | 22.0 (18.1-25.5) | **0.001** |
| % Age 18-29 | 13.2 (10.0-16.8) | 14.8 (12.2-18.5) | **<0.001** |
| % Age 30-44 | 18.5 (15.1-21.8) | 18.0 (14.8-21.2) | 0.132 |
| % Age 45-64 | 27.7 (24.5-31.2) | 25.7 (21.8-29.1) | **<0.001** |
| % Age over 65 | 16.7 (13.0-22.4) | 17.7 (14.0-21.7) | **0.013** |
| % Male | 49.0 (47.0-51.5) | 48.1 (45.0-51.0) | **<0.001** |
| % Non-white | 28.4 (16.1-46.4) | 39.3 (23.9-58.4) | **<0.001** |
| % Hispanic | 5.9 (3.8-9.4) | 4.0 (2.3-6.7) | **<0.001** |
| SVI | 0.3 (0.1-0.6) | 0.6 (0.4-0.8) | **<0.001** |
| Income (×$1000) | 68.6 (55.9-88.0) | 46.6 (37.1-56.9) | **<0.001** |
| % Unemployed | 4.1 (2.2-6.2) | 5.2 (3.0-8.3) | **<0.001** |
| PCP rate | 0.2 (0.0-0.6) | 0.4 (0.1-1.1) | **<0.001** |
| Hospitals | 0.0 (0.0-1.0) | 0.1 (0.0-1.0) | **<0.001** |
| % Uninsured | 10.4 (7.9-12.6) | 10.8 (9.0-12.1) | 0.063 |
| Mortality rate | 43.7 (36.0-53.0) | 56.0 (47.4-64.2) | **<0.001** |
| % in Poverty | 8.2 (5.3-12.1) | 13.5 (9.7-17.3) | **<0.001** |
| % in Rural | 7.6 (1.4-28.1) | 51.2 (17.8-82.6) | **<0.001** |
| COVID-19 ED visits | 1,478 (904-2,349) | 953 (515-1,635) | **<0.001** |
| COVID-19 deaths | 69 (43-104) | 56 (26-90) | **<0.001** |

**Table G**: Characteristics of census tracts that are projected to have high (groups: 20-49, 50-99, 100-249, and 250-399) and low (group: 0-19) MHC utilization for COVID-19 vaccination any time during 12-month period. Median values with IQR and p-values for significance of the difference of medians are provided.

|  | **High Utilization**  **N = 382** | **Low Utilization**  **N = 914** | **P-value** |
| --- | --- | --- | --- |
| % Age under 18 | 21.3 (17.3-25.5) | 21.6 (18.1-24.9) | 0.472 |
| % Age 18-29 | 13.1 (9.9-16.0) | 14.6 (11.9-18.4) | **<0.001** |
| % Age 30-44 | 18.9 (14.9-22.4) | 17.9 (14.9-21.0) | **0.008** |
| % Age 45-64 | 26.8 (23.1-30.1) | 26.4 (22.7-29.8) | 0.267 |
| % Age over 65 | 17.3 (13.3-22.9) | 17.3 (13.5-21.4) | 0.894 |
| % Male | 48.7 (46.3-51.2) | 48.4 (45.8-51.2) | 0.190 |
| % Non-white | 35.7 (22.2-57.4) | 34.6 (19.6-52.0) | 0.587 |
| % Hispanic | 5.2 (3.1-8.1) | 4.6 (2.7-7.5) | **0.043** |
| SVI | 0.4 (0.1-0.7) | 0.6 (0.3-0.8) | **<0.001** |
| Income (×$1000) | 69.6 (53.2-91.3) | 50.4 (39.2-62.6) | **<0.001** |
| % Unemployed | 4.5 (2.3-7.1) | 4.7 (2.7-7.6) | **<0.001** |
| PCP rate | 0.2 (0.0-0.6) | 0.4 (0.1-1.0) | **<0.001** |
| Hospitals | 0.0 (0.0-0.4) | 0.2 (0.0-1.0) | **<0.001** |
| % Uninsured | 9.6 (7.3-12.0) | 10.9 (9.0-12.4) | **<0.001** |
| Mortality rate | 44.6 (35.3-53.9) | 53.6 (44.6-62.6) | **<0.001** |
| % in Poverty | 8.1 (4.8-11.8) | 12.7 (9.2-16.5) | **<0.001** |
| % in Rural | 15.0 (1.8-49.3) | 34.0 (6.9-64.3) | **<0.001** |
| COVID-19 ED visits | 1,155 (627-1,999) | 1,151 (661-2,005) | 0.951 |
| COVID-19 deaths | 54 (29-87) | 66 (36-101) | **<0.001** |
